# *N*-Acetylcysteine as a Bacterial Antibiofilm Adjuvant: Mechanisms, Synergistic Combinations and Clinical Translation

**DOI:** 10.3390/life16091414

**Published:** 2026-08-26

**Authors:** Anastasia N. Golub, Natalia N. Mikhailova, Maria V. Pomytkina, Ksenia V. Eremeeva, Elena A. Shevchik, Galina N. Nikiforova, Valeriy M. Svistushkin, Vera V. Korennaya, Yuriy L. Vasil’ev, Elena O. Bakhrushina

**Affiliations:** 1 Department of Pharmaceutical Technology, A.P. Nelyubin Institute of Pharmacy, I.M. Sechenov First Moscow State Medical University (Sechenov University), 119048 Moscow, Russia; 2 Department of Biological Chemistry, Institute of Digital Biodesign and AI in Medicine, I.M. Sechenov First Moscow State Medical University (Sechenov University), 119048 Moscow, Russia; 3 Department of Ear, Throat and Nose Diseases, N.V. Sklifosovsky Institute of Clinical Medicine, I.M. Sechenov First Moscow State Medical University (Sechenov University), 119048 Moscow, Russia; 4 Department of Obstetrics and Gynecology, Faculty of Pediatrics, Russian Medical Academy of Continuing Professional Education, 125993 Moscow, Russia; 5 Moscow Clinical Scientific and Research Center Hospital 52 of the Moscow Health Department, 123182 Moscow, Russia; 6 Department of Operative Surgery and Topographic Anatomy, N.V. Sklifosovsky Institute of Clinical Medicine, I.M. Sechenov First Moscow State Medical University (Sechenov University), 119048 Moscow, Russia

**Keywords:** *N*-acetylcysteine, bacterial biofilms, antibiofilm activity, synergy with antibiotics, mechanisms of action, drug delivery systems

## Abstract

*N*-acetylcysteine (NAC) is a synthetic derivative of L-cysteine, known since the mid-20th century as a mucolytic agent and, in recent decades, has attracted attention for its antioxidant and antibiofilm properties. Bacterial biofilms are structured communities of microorganisms enclosed in an extracellular polymeric matrix, which accounts for their markedly increased resistance to antibiotics (up to 1000-fold higher than in planktonic forms) and to the host immune response. According to the literature, up to 65% of infectious agents are associated with biofilm formation, making them a challenging therapeutic target. This review systematizes current data on the molecular mechanisms of the antibiofilm action of NAC, including disruption of matrix proteins and polysaccharides, degradation of extracellular DNA, suppression of the quorum sensing system, and disturbance of bacterial redox homeostasis. Particular attention is given to synergistic combinations of NAC with antibiotics of five major classes; effective concentrations are provided, and the types of interaction are characterized. The results of clinical studies from the last decade are reviewed, demonstrating the potential of NAC as an adjuvant in urinary tract infections, chronic rhinosinusitis, diabetic osteomyelitis, and cystic fibrosis. The main limitations (pH dependence, instability, low oral bioavailability) are critically evaluated, and approaches to overcoming them using nanoparticles, hydrogels, and combinations with propolis or chitosan are proposed. The review is intended for researchers in antimicrobial chemotherapy and developers of new drug delivery systems.

## 1. Introduction

N-acetyl-L-cysteine (NAC) was first synthesized in 1950 by Professor Vittorio Ferrari. Its ability to cleave disulfide bonds provided the basis for its use as a mucolytic agent. A patent for the compound was obtained in 1960, and clinical use began in 1967 [1]. NAC was initially used in cystic fibrosis (from 1969), after which its range of application expanded to the treatment of chronic obstructive pulmonary disease.

A substantial body of data on the antibiofilm potential of NAC has been accumulated by now. In 2016, a key systematic review was published on the role of NAC in the treatment of biofilm-associated respiratory infections [2]. However, it addressed exclusively respiratory diseases, without considering urinary tract infections, wound infections, or other localizations. Moreover, combinations with antibiotics were not analyzed. More recent reviews [3,4] do not delve into the details of molecular mechanisms and do not provide a critical evaluation of clinical data. This review fills these gaps by offering a comprehensive account of the mechanisms, synergistic interactions, clinical studies, and limitations, as well as promising ways of overcoming the shortcomings of NAC, including a clinically oriented analysis of delivery.

In recent years, alternative strategies to counter biofilm-associated infections have been actively developed, including antimicrobial peptides (AMPs) with selective or pH-responsive activity [5], such as the pH-sensitive peptide GH12, which selectively suppresses cariogenic bacteria while supporting commensal flora [6]; synthetic cationic small molecules (e.g., quaternary phosphonium salts) [7]; and delivery systems that enhance the penetration of antimicrobial agents into biofilms (liposomes, nanoparticles) [8]. These approaches demonstrate efficacy against resistant pathogens, but often require complex design and do not always disrupt mature biofilms. Against this background, *N*-acetylcysteine (NAC) stands out due to its multitargeted mechanism, which includes matrix degradation, suppression of quorum sensing, and disruption of redox balance, allowing it to act on already formed biofilms and synergistically enhance antibiotics. However, its clinical application is limited by pH dependence, instability, and low bioavailability—the same challenges that are being addressed through innovative delivery systems actively explored for other antimicrobial agents [5,6,7,8]. This makes NAC a promising candidate for adjuvant therapy, provided that optimal dosage forms are developed.

## 2. Materials and Methods

This review is narrative (non-systematic) and was carried out to summarize current data on the mechanisms of the antibiofilm action of NAC, its synergy with antimicrobial agents, and clinical prospects. The methodological approach included the search, selection, evaluation, and synthesis of relevant literature in the following areas: the epidemiological significance of biofilms and the mechanisms of their formation, the chemical properties of NAC, mechanisms of antibiofilm action (matrix disruption, suppression of adhesion, effects on quorum sensing and redox balance), synergistic combinations with antibiotics, clinical studies and limitations, and promising dosage forms.

### 2.1. Literature Search Strategy

The search was conducted in the PubMed, Web of Science, and Google Scholar databases. In addition, the reference lists of key systematic reviews on the topic were screened manually to identify additional relevant publications. The main period covers publications from 1997 to 2026, since the first studies on the antibiofilm activity of NAC appeared in 1997. The following keywords and their combinations were used in English: “*N*-acetylcysteine,” “NAC,” “biofilm,” “antibiofilm activity,” “biofilm eradication,” “matrix disruption,” “quorum sensing,” “synergy,” “antibiotics,” “combination therapy,” “adjuvant therapy,” “chronic infection,” “clinical trial,” “Pseudomonas aeruginosa,” “Staphylococcus aureus,” “Escherichia coli.” Boolean operators AND/OR were used to broaden the search.

### 2.2. Inclusion and Exclusion Criteria

Inclusion criteria: the review included original experimental studies (in vitro, ex vivo, in vivo in animals), clinical studies (randomized controlled, prospective cohort, retrospective, pilot), systematic reviews and meta-analyses, as well as individual full-text review articles in English containing primary data or summaries on the topic. Selection was based on the presence of data on the effect of NAC on biofilm formation or disruption; a description of the mechanisms of antibiofilm action; experimental or clinical data on the efficacy of NAC as monotherapy or in combination with antibiotics; and information on minimum inhibitory concentrations (MICs) or concentrations effective against biofilms.

Exclusion criteria: publications not related to NAC or biofilms; studies devoted exclusively to the mucolytic action of NAC without analysis of antibiofilm properties; review articles, letters, and editorials without primary data; duplicate publications; and sources for which the full text was unavailable or that provided insufficient information for analysis.

### 2.3. Source Selection and Systematization

Initial selection was performed by title, abstract, and keywords. Full texts were analyzed at the second stage. Sources were grouped by the thematic sections of the review. For included studies, the following data were extracted: microorganism/pathogen, NAC concentration, effect (inhibition of formation, disruption of mature biofilm, synergy with antibiotics), type of antibiotic (for combinations), clinical outcome (if available), and limitations.

A meta-analysis was not performed owing to the heterogeneity of study designs and differences in experimental conditions. Formal quality assessment using the GRADE, PRISMA, or Cochrane Risk of Bias tools was not applied, consistent with narrative review methodology. However, study design, sample size, presence of controls, stage of clinical development, and completeness of methods description were taken into account when interpreting the data. Priority was given to randomized controlled trials, prospective clinical studies, and original full-text articles.

## 3. Relevance of Combating Biofilms

### 3.1. Definition and Epidemiological Significance

A biofilm is a three-dimensional structured community of microbial cells enclosed in a self-produced polymeric matrix attached to biotic or abiotic surfaces. A biofilm may contain one or several species of microorganisms, including bacteria, fungi, protozoa, and viruses [9]. Biofilms play a key role in the development of resistance to antibiotics (up to 1000-fold higher than in planktonic cells) and to the host immune response, contributing to the chronicity of infections. According to the literature, the majority (65%) of infectious agents are associated with biofilms and exhibit high resistance to antimicrobial agents [10].

### 3.2. Mechanism of Biofilm Formation

**Figure 1:**
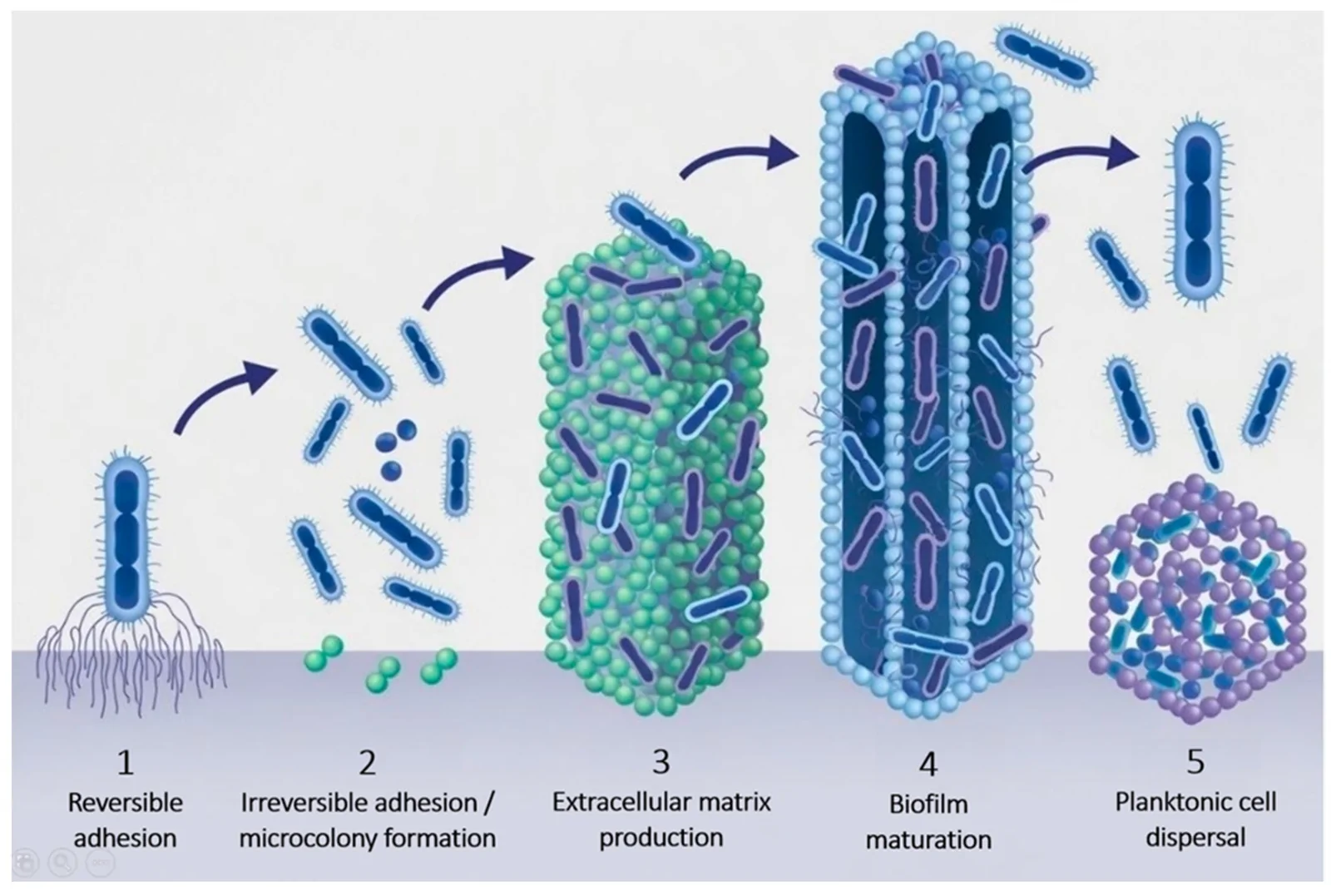
Biofilm formation process.

Biofilm formation is a multistage process. In the first stage, reversible adhesion of planktonic bacteria to a surface occurs via surface structures (pili, flagellins, adhesins). This is followed by the formation of microcolonies and production of extracellular matrix components (exopolysaccharides, proteins, extracellular DNA), which gives the biofilm its three-dimensional structure. In the final stage, the biofilm may disperse, releasing planktonic cells to colonize new niches. Each of these stages can serve as a target for antibiofilm agents (Figure 1, based on [2]).

### 3.3. Biofilms on Medical Devices

Biofilms forming on the surface of implantable medical devices (catheters, prostheses, mechanical ventilators, pacemakers) are of particular clinical significance. The incidence of device-associated infections ranges from 2% for joint prostheses to 40% for mechanical circulatory support devices [11]. The high incidence of such infections underscores the need to develop effective antibiofilm strategies, including the use of compounds capable of preventing implant colonization.

### 3.4. Main Biofilm-Forming Pathogens and Associated Infections

Table 1 presents the most clinically significant microorganisms and typical sites of infection. The most common causative agents are *Pseudomonas aeruginosa*, *Staphylococcus aureus*, and *Escherichia coli*, and the same microorganism may cause different diseases depending on localization. Biofilms can form both on medical devices and directly in body tissues, underscoring the need for universal antibiofilm approaches.

The data presented indicate a wide range of microorganisms capable of forming biofilms across various anatomical sites—from the respiratory and urinary tracts to wound surfaces and implanted devices. The same pathogen (e.g., *S. aureus* or *P. aeruginosa*) can cause infections in different locations, highlighting the universal nature of the biofilm survival mechanism. At the same time, the aetiological structure varies significantly depending on the clinical scenario, complicating the development of a unified therapeutic approach. In this context, adjuvant agents with broad-spectrum antibiofilm activity that can act against diverse microbial species and their extracellular matrices are of particular interest. One such promising compound is *N*-acetylcysteine, the mechanisms and clinical potential of which are reviewed here.

### 3.5. Current Approaches to Combating Biofilms

Current strategies fall into two main groups: prevention of formation and disruption of already established biofilms. Prevention aims to suppress initial adhesion by modifying surfaces and inhibiting the expression of adhesins, exopolysaccharide synthesis, and quorum sensing signaling. Destructive approaches include the use of enzymes (DNases, proteases), chelating agents, antimicrobial peptides, and nanoparticles, as well as physical methods (ultrasound, electric and magnetic fields, photodynamic therapy) and biological methods (bacteriophages, probiotics) [4]. Each method has limitations, which stimulates the search for new adjuvant compounds, in particular NAC.

## 4. NAC: From Mucolytic to Antibiofilm Agent

### 4.1. Chemical Structure and Properties

**Figure 2:**
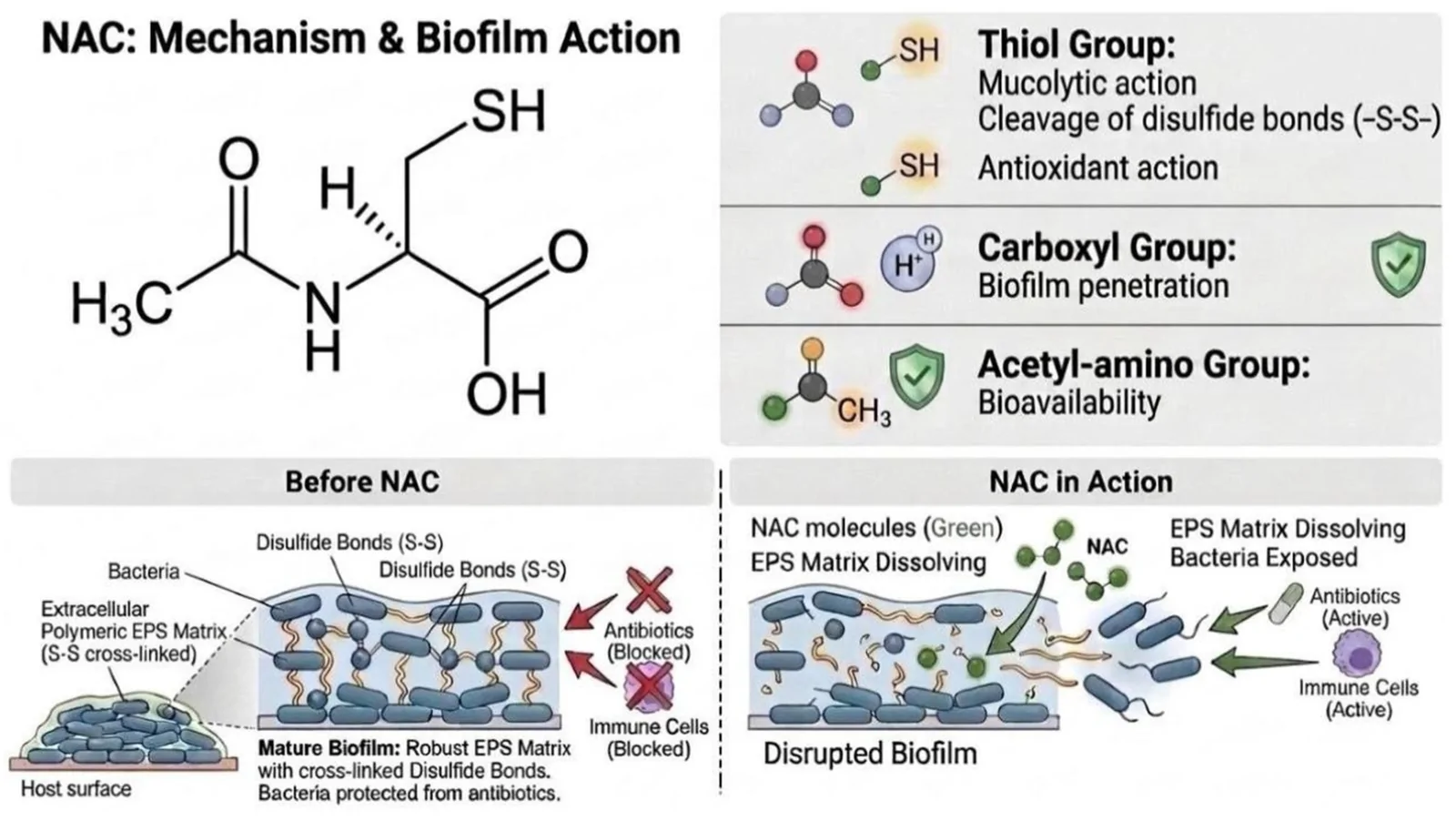
Chemical structure and mechanism of the antibiofilm action of acetylcysteine.

N-acetyl-L-cysteine is a synthetic derivative of the natural amino acid L-cysteine and belongs to the class of thiols. Its structure contains a thiol group (–SH), which confers antioxidant and mucolytic properties; a carboxyl group, which determines its acid–base properties; and an acetylated amino group, which increases stability and bioavailability compared with cysteine (Figure 2).

The physicochemical properties of NAC, including pKa, stability, susceptibility to oxidation, solubility, and pharmacokinetics, and their impact on pharmaceutical formulation development and therapeutic efficacy, are discussed further in Section 8.

### 4.2. First Studies of Antibiofilm Activity

The first studies on the effect of NAC on biofilm formation appeared in 1997. In 2000–2004, research began into the mechanisms of inhibition of biofilm formation; in 2005–2009, papers appeared on biofilms on medical devices and the safety of NAC. In 2010–2014, there was an increase in clinical studies and research into synergy with antibiotics, along with the publication of the first systematic review (2014). Since 2015, the mechanisms of biofilm disruption have been studied in greater depth, and the range of pathogens investigated has expanded.

A key study from this period is that of Zhao and Liu [13], which showed that NAC at a concentration of 0.5 mg/mL disrupts mature *P. aeruginosa* biofilms, with complete eradication achieved at 10 mg/mL. Synergy with ciprofloxacin was observed in only 50% of cases. The review by Blasi et al. [2] summarized data on the use of NAC in respiratory infections, and the study by Manoharan et al. [14] demonstrated that combining NAC with antibiotics leads to disruption of ≥90% of *S. aureus* biofilms. The recent study by Sainglers et al. [12] found that NAC (15 mM) suppresses the formation of neutrophil extracellular traps and reduces the biomass of *B. pseudomallei* biofilms.

### 4.3. Rationale for the Use of NAC Against Biofilms

The rationale for using NAC as an antibiofilm agent is based on the similarity between the biofilm extracellular matrix and mucin, in which NAC cleaves disulfide bonds. In vitro studies confirm that NAC not only inhibits the formation of new biofilms but also disrupts mature ones, facilitating antibiotic access to bacterial cells [2,13].

## 5. Mechanisms of the Antibiofilm Action of NAC

The antibiofilm activity of *N*-acetylcysteine (NAC) is multifactorial and includes effects on extracellular polymeric substances (EPS), suppression of bacterial adhesion, modulation of quorum sensing (QS), regulation of oxidative stress and thiol–disulfide exchange, changes in bacterial metabolism, and modulation of the host inflammatory response. The magnitude and direction of these effects depend on NAC concentration, environmental pH, the bacterial species involved, the stage of biofilm development, and the experimental model.

### 5.1. Effects on Extracellular Polymeric Substances (EPS) and Disruption of the Extracellular Matrix

The biofilm extracellular matrix contains proteins, polysaccharides and extracellular DNA (eDNA). NAC can interfere with components of this matrix: it affects matrix proteins and DNA, reduces extracellular polysaccharide production, and can promote DNA cleavage, contributing to biofilm destabilization [14,15,16]. Disulfide bonds are known to contribute to the stability or functional conformation of several extracellular proteins of *P. aeruginosa*, including the amyloid protein FapC, lipase, and elastase; however, direct reduction of disulfide bonds in these proteins by NAC has not been experimentally demonstrated [17]. With respect to polysaccharides, NAC can alter biofilm architecture by reducing EPS production. Olofsson et al. [15] reported decreased production of extracellular polysaccharides in several bacterial isolates, including at concentrations that did not affect bacterial growth. In *P. aeruginosa*, 0.5 and 1 mg/mL NAC reduced EPS production by approximately 27.6% and 44.6%, respectively. At 10 mg/mL, complete disruption of mature biofilms was observed in this model [13]. In a chronic wound biofilm model, 4 mg/mL NAC inhibited biofilm formation, whereas 10–20 mg/mL markedly disrupted established biofilms; at 20 mg/mL, substantial biofilm disruption was observed within 24 h. At higher concentrations, NAC also reduced the amount of detectable eDNA [16]. The effect of NAC on the matrix therefore includes reduced EPS production, destabilization of mature biofilms, and a decrease in eDNA content.

The effects of NAC on the biofilm matrix include reduced EPS production, destabilization of mature biofilms, and a reduction in detectable eDNA. Importantly, these effects are pH-dependent. In mucoid *P. aeruginosa* biofilms, NAC penetrated the matrix and exerted bactericidal activity when the environmental pH was below its pKa (~3.3), favoring the undissociated form of NAC [18].

Bacterial killing was accompanied by microcolony swelling and cell release, while the residual matrix remained intact and displayed pH-responsive hydrogel behavior. Thus, pH appears to modulate NAC penetration and biofilm destabilization rather than directly causing matrix degradation.

The reducing activity of NAC toward disulfide bonds may contribute to disruption of the protein component of the biofilm matrix by altering the structure of extracellular or surface-associated proteins and, consequently, matrix organization and bacterial adhesion. However, direct reduction by NAC of specific disulfide bonds in protein targets within mature *P. aeruginosa* biofilms has not yet been demonstrated. Thus, thiol–disulfide exchange remains a plausible, but not yet fully established, molecular mechanism of NAC antibiofilm activity.

### 5.2. Suppression of Adhesion and Initial Stages of Formation

NAC interferes with the early stages of biofilm formation by reducing bacterial attachment and aggregation. Surface proteins, pili, and flagellar structures are involved in adhesion, and some of these proteins contain disulfide bonds; their reduction has been proposed as one possible mechanism. However, the anti-adhesive effect of NAC is unlikely to be explained by this mechanism alone, as NAC also affects EPS, bacterial surface properties, and motility. In the bacterial isolates examined, NAC reduced the number of attached cells by 51–99%, depending on the strain and incubation time [15]. In *P. aeruginosa*, 20–30 mM NAC completely suppressed bacterial attachment to the substrate and also reduced aggregation and other features associated with biofilm formation [19].

### 5.3. Effect on the Quorum Sensing (QS) System

Quorum sensing is a mechanism of intercellular communication based on the production and perception of autoinducers, whose concentration correlates with population density. QS regulates the expression of virulence genes and biofilm formation [20]. LasR and RhlR are two major regulatory receptors of the quorum sensing (QS) system in *P. aeruginosa*, controlling the expression of virulence genes and biofilm formation, while AHLs (N-acylhomoserine lactones) act as signaling autoinducers [21,22]. Lima et al. [21] showed that NAC can modulate QS-dependent processes in *P. aeruginosa*. Molecular docking suggested that NAC may occupy the ligand-binding regions of LasR and RhlR like natural AHLs. At 125 μM, pyocyanin and rhamnolipid production decreased by up to 34% and 37%, respectively. However, the direct anti-QS activity of native NAC is moderate. Selected hybrid N-acylcysteines containing acyl groups derived from benzoic acid derivatives showed more pronounced anti-QS and antibiofilm activity than standard NAC in experimental models [23]. At the same time, disruption of *P. aeruginosa* biofilms by NAC can occur without detectable changes in several QS-regulated genes and products. Huang et al. [24] reported a reduction in mature biofilm biomass without changes in some of the QS-regulated genes examined.

This finding points to the involvement of QS-independent redox mechanisms, discussed in more detail in Section 5.4. Thus, QS modulation may contribute to the overall antibiofilm effect of NAC but does not appear to fully account for it.

### 5.4. Regulation of Intracellular Redox Balance and Oxidative Stress

Changes in bacterial redox balance can substantially affect biofilm formation and stability [24]. The redox activity of NAC is not limited to its effects on reactive oxygen species (ROS). Its thiol group can also participate directly in thiol–disulfide exchange, altering the redox state of cysteine residues in proteins independently of ROS scavenging [25,26]. The effects of NAC should therefore not be explained solely by direct scavenging of ROS. Their direction depends on NAC concentration, pH, bacterial species, and experimental conditions. The effects of NAC on bacterial redox homeostasis should also be considered in the context of species-specific systems of low-molecular-weight thiols. Different bacterial groups use glutathione, bacillithiol, mycothiol, and related compounds to maintain intracellular redox homeostasis and protect protein thiols from oxidation [27,28]. In a chronic wound biofilm model, NAC caused a dose-dependent decrease in the NAD^+^/NADH ratio. At concentrations of 5, 7.5, 10, and 20 mg/mL, the NAD^+^/NADH ratio decreased by 43.3%, 56.8%, 75.4%, and 84.1%, respectively, compared with control, indicating increased oxidative stress. In this model, high NAC concentrations were associated with a marked shift in intracellular redox and metabolic status, increased oxidative stress, and inhibition of protein synthesis [16].

Under other conditions, NAC has the opposite effect and acts as an antioxidant. In *E. coli*, NAC reduced intracellular ROS induced by subinhibitory ciprofloxacin concentrations by approximately 40%, decreased SOS-response induction by up to 75%, and suppressed SOS-dependent mutagenesis [29]. In mature *P. aeruginosa* biofilms, ROS scavenging with 1 mM NAC also reduced biofilm biomass by disrupting redox-dependent mechanisms involved in maintaining cooperative behavior within the bacterial population [24].

### 5.5. Effects on Bacterial Metabolism

NAC can alter bacterial metabolism by affecting energy metabolism, amino acid and sulfur-containing compound metabolism, as well as redox cofactors.

In *P. aeruginosa*, NAC suppresses the denitrification pathway and associated anaerobic respiratory processes and also reduces flagellum-mediated motility [30]. Because denitrification is important for the adaptation of *P. aeruginosa* to oxygen-limited conditions, this effect may also be relevant to hypoxic regions of mature biofilms. However, this specific adaptation was not directly measured by Valzano et al. [30], and should therefore be regarded as a physiologically plausible interpretation. Studies in other bacterial species show that the metabolic effects of NAC are not limited to respiratory pathways. In *Xanthomonas citri*, a plant pathogen that is not a human pathogen, NAC altered cysteine and nitrogen metabolism and changed the intracellular levels of several proteinogenic amino acids [31]. In *Edwardsiella tarda*, primarily an aquatic and animal pathogen and not a typical human pathogen, NAC activated amino acid metabolic pathways, increased intracellular glutathione levels, and reduced oxidative stress; however, these changes were accompanied by increased resistance to doxycycline and several other antibiotics [32]. Thus, the metabolic effects of NAC may, under some conditions, be associated with reduced antibiotic susceptibility.

The metabolic effects of NAC include changes in respiratory pathways, redox cofactors, and amino acids and cannot be attributed solely to its antioxidant properties.

### 5.6. Modulation of the Host Immune Response During Biofilm-Associated Infections

In addition to its direct effects on bacteria, NAC can modulate the host inflammatory response, thereby influencing tissue damage, immune cell function, and the local conditions that contribute to biofilm persistence.

In a rat model of *P. aeruginosa* pneumonia, NAC reduced lung injury without a corresponding decrease in bacterial burden, suggesting a contribution of host-response modulation alongside its direct antibacterial effects. This was accompanied by changes in the regulation of inducible and endothelial nitric oxide synthases (iNOS and eNOS) [33]. However, this model represents an acute bacterial infection rather than a mature biofilm-associated infection.

NAC also affects cytokine production by innate immune cells. In LPS-activated macrophages, 15 mM NAC reduced the expression and secretion of TNF-α, IL-1β, and IL-6, while also decreasing IL-10 [34]. Since both pro- and anti-inflammatory mediators were affected, this response is better described as a modulation of the cytokine profile than as simple suppression of inflammation.

Another aspect of NAC-mediated immunomodulation involves neutrophil extracellular traps (NETs). In a *Burkholderia pseudomallei*–neutrophil co-culture model, NET formation was associated with increased biofilm formation, whereas 15 mM NAC inhibited infection-induced NET formation, reduced NET-associated biofilm formation, and simultaneously enhanced neutrophil-mediated bacterial killing through phagocytosis and degranulation [12].

Because the same concentration of NAC also exerted direct antibacterial activity, the reduction in biofilm formation cannot be attributed solely to NET inhibition.

The effects of NAC on macrophage polarization appear to depend strongly on the experimental setting. In LPS-stimulated macrophages, the initial reduction in ROS induced by NAC was followed by compensatory activation of a signaling pathway that could enhance oxidative stress and sustain M1 polarization [35]. In contrast, in a model of LPS-induced intrauterine inflammation, targeted delivery of NAC as a dendrimer conjugate (DNAC) reduced pro-inflammatory markers and shifted the M1/M2 macrophage balance toward an M2-like phenotype [36]. These findings relate to DNAC rather than free NAC and were obtained outside the setting of a mature biofilm-associated infection.

The immunomodulatory effects of NAC are also linked to oxidative stress in host tissues. In a diabetic rat wound model, topical and systemic NAC reduced markers of oxidative damage and improved wound healing [37]. Although biofilm formation was not specifically examined in this model, the findings support a role for NAC in tissue repair.

More direct evidence relevant to biofilm-associated wounds comes from a chronic wound model in diabetic mice, in which NAC combined with α-tocopherol reduced oxidative stress and bacterial biofilm burden and was associated with restoration of granulation tissue and more normal collagen deposition and remodeling [38]. Because NAC was administered together with α-tocopherol, the overall reparative effect cannot be attributed to NAC alone.

Host immune modulation should be considered an indirect component of the antibiofilm effect of NAC. In addition to its direct effects on the biofilm matrix and bacterial cells, NAC can reduce excessive inflammation and oxidative stress, limit NET formation and tissue damage, and support phagocytic clearance and tissue repair. These host-mediated effects may further reduce conditions that favor chronic biofilm persistence.

### 5.7. Comparison with Other Antibiofilm Agents

Table 2 presents a comparison of NAC with other chemical antibiofilm agents in terms of strengths and weaknesses. NAC occupies a unique position owing to its multiple mechanisms of action and the absence of direct resistance at low doses, although it is inferior to some agents in terms of stability and pH dependence.

## 6. Combined Use of NAC with Antibiotics

The main limitation of antibiotic therapy in biofilm-associated infections is the low permeability of the matrix to antibiotics. By disrupting the matrix, NAC facilitates drug penetration into deeper layers, thereby reducing the minimum inhibitory concentration (MIC) and increasing efficacy.

### 6.1. Combinations with β-Lactams

NAC exhibits synergy with certain β-lactams. In the study by De Angelis et al. [39], combining NAC (2.5 mg/mL) with meropenem reduced the MIC of the antibiotic for carbapenem-resistant *Klebsiella pneumoniae* from 256–1024 µg/mL to 8–32 µg/mL. For *A. baumannii*, synergy was shown with ampicillin/sulbactam, whereas antagonism was noted with imipenem and polymyxins [39]. The combination with cefditoren is active against *S. pneumoniae* biofilms: adding 2.5 mg/mL NAC to low doses of cefditoren reduced biofilm biomass by >80% and adhesion to lung cells by 40%; a similar effect was not observed with cefixime [40].

### 6.2. Combinations with Aminoglycosides

NAC potentiates the action of a limited number of aminoglycosides. A combination of 2500–1.07 µM NAC with tobramycin suppressed *P. aeruginosa* biofilm formation by 63.5% [21].

### 6.3. Combinations with Polymyxins

Valzano et al. [30] showed that significant synergy is observed when NAC (8000 mg/L) is added to colistin (8 mg/L), providing complete eradication of *P. aeruginosa* biofilms regardless of resistance phenotype.

### 6.4. Combinations with Tetracyclines

The study by Feng et al. [41] in *A. baumannii* showed that the combination of 4 mg/mL NAC and 0.5 µg/mL tigecycline exhibits synergy, while 16 mg/mL NAC or 2 µg/mL tigecycline alone provides a significant bactericidal effect.

### 6.5. Combinations with Macrolides

A liposomal formulation of azithromycin with NAC (LAN) showed a reduction in the MIC for *E. coli* from 16 to 2.5 µg/mL (6.4-fold) and disruption of the biofilm by up to 93.2% with low cytotoxicity; however, LAN is unstable in plasma and sputum [42].

Summary data on the synergy of NAC with antibiotics are presented in Table 3.

Thus, NAC exhibits synergy with antibiotics from most major classes, most notably with β-lactams (meropenem, ampicillin/sulbactam, cefditoren), polymyxins (colistin), tetracyclines (tigecycline), macrolides (azithromycin), and fosfomycin. However, the efficacy of combinations varies considerably: in some cases, complete biofilm eradication is observed; in others, only partial effects are observed, and antagonism has been reported with certain antibiotics (e.g., imipenem). This indicates strain- and antibiotic-specific synergy, necessitating careful selection of combinations in clinical practice. Nevertheless, the ability of NAC to reduce minimum inhibitory concentrations (MICs) of antibiotics opens prospects for lowering toxicity and slowing resistance development, which is particularly important in the treatment of chronic biofilm-associated infections. The presented data form the basis for clinical evaluation of NAC as an adjuvant, which is discussed in the next section.

## 7. Clinical and Preclinical Data

### 7.1. Key Clinical and Preclinical Studies

Early studies (1997–2016) are summarized in the systematic review by Blasi et al. [2], which compiled data on the minimum inhibitory concentrations (MICs) of NAC for a wide range of pathogens, including *E. coli*, *P. aeruginosa*, and *S. aureus.* MIC values were found to vary widely from 0.003 to 80 mg/mL depending on the microorganism species, strain, and experimental conditions. The lowest MICs were noted for certain Gram-positive cocci and anaerobes, whereas higher concentrations were required for *P. aeruginosa* and *A. baumannii.* Over the past decade (2016–2026), studies have appeared evaluating the antibiofilm effect of NAC directly in patients.

A summary of the most significant clinical and preclinical studies is presented in Table 4 and Table 5, respectively.

This review considers 7 unique clinical studies, involving a total of 380 to 400 participants.

The findings are encouraging, but their results should be interpreted with caution, for the following reasons:

(1) Heterogeneity of designs: Only three studies are RCTs [45,46,48], of which only one [45] is open-label, while the other two [46,48] are randomized but also without placebo or blinding. The remaining studies are observational or pilot in nature, which reduces the level of evidence.
(2) Diversity of pathologies and treatment regimens: NAC was used for various infections (prostatitis, rhinosinusitis, osteomyelitis, *H. pylori*, catheter-related infections, respiratory tract infections), at different doses (oral 600 mg/day or 600 mg twice daily, topical from 2.5 to 49.6 mg/mL), and in combination with various antibiotics (fosfomycin, standard regimens, thiamphenicol). This precludes the development of a single optimal regimen and complicates direct comparison.
(3) Combination therapy: In all studies, NAC was administered together with other agents (antibiotics or symptomatic treatments). The observed positive effect (reduced recurrence, accelerated healing, eradication) cannot be unambiguously attributed to the antibiofilm action of NAC alone, since it also possesses mucolytic, anti-inflammatory, and antioxidant activity. Without monotherapy or the use of in vivo biofilm surrogate markers, it is impossible to isolate the contribution of NAC.
(4) Lack of direct biofilm assessment: No study performed quantitative visualization of biofilms in patient tissues (for example, confocal microscopy of biopsies, fluorescent probing). Outcomes were assessed using indirect clinical signs (CRP level, healing time, recurrence rate), which are nonspecific to the biofilm process.
(5) Small samples and short-term follow-up: Cohort sizes ranged from 40 to 102 patients, and the follow-up period in most studies did not exceed 3–6 months. For chronic biofilm-associated infections prone to recurrence, this is insufficient to judge long-term efficacy and safety.
(6) Time span: The studies span the period from 2006 to 2026, which reflects the evolution of approaches but also introduces additional heterogeneity (changes in diagnostic and treatment standards).

The data obtained from these studies indicate the potential usefulness of NAC as an adjuvant in biofilm-associated infections, particularly in urology and ENT practice. However, the evidence base remains moderate (low/moderate level on the GRADE scale). Definitive recommendations will require large, multicenter, double-blind, placebo-controlled RCTs using objective methods of biofilm assessment (molecular imaging, microbiological analysis of biopsies) and standardized combination therapy protocols. For now, the clinical data should be regarded as encouraging but requiring confirmation.

Preclinical studies convincingly demonstrate the antibiofilm properties of NAC in vitro and in animal models, providing biological rationale for clinical trials. However, they cannot serve as direct evidence of clinical efficacy owing to methodological limitations. Their main value lies in elucidating mechanisms, optimizing concentrations, and developing delivery systems.

Translation into clinical practice will require standardized preclinical protocols, testing on clinical isolates, and, most importantly, randomized controlled trials in humans using objective biofilm markers.

### 7.2. Key Trends in Clinical Application

Analysis of published data identifies the following trends. Adding oral NAC (600 mg/day) to fosfomycin in chronic bacterial prostatitis achieved complete pathogen eradication in 75% of patients [43]. In another study, NAC completely prevented biofilm formation on urethral stents [48]. Irrigation with NAC at concentrations of 3.1–49.6 mg/mL in chronic rhinosinusitis effectively inhibited the formation and disrupted mature *S. aureus* biofilms [44]. A preclinical study in mice showed that a hydrogel dressing containing NAC accelerates the healing of infected wounds [54]. Adding 600 mg of NAC twice daily to standard therapy for diabetic foot osteomyelitis shortened the time to clinical response and lowered C-reactive protein levels [45].

## 8. Promising NAC Delivery Strategies: From Biopharmaceutical Limitations to Clinical Solutions

Despite its considerable potential as an antibiofilm agent, the clinical application of NAC is limited by a number of fundamental biopharmaceutical problems.

NAC contains two main ionizable groups: a carboxyl group with a pKa of approximately 3.3 and a thiol group with a pKa of approximately 9.87; consequently, the molecule predominantly exists in a monoanionic form within the range 3.3 < pH < 9.87 [18,22]. In several experimental models, its antibiofilm activity is most pronounced at pH values below pKa ≈ 3.3, which has been attributed to the pH-dependent behavior of NAC as a weak acid, its penetration into the biofilm matrix, and subsequent bactericidal activity [16,18]. At physiological pH values (7.2–7.4), the efficacy of NAC is significantly reduced, which limits its systemic use and requires the development of local dosage forms that maintain an acidic microenvironment or the use of buffer systems. In addition, disrupting mature *P. aeruginosa* biofilms requires NAC concentrations ≥10 mg/mL, and complete eradication requires 20 mg/mL [16], which may be difficult to achieve with systemic administration owing to bioavailability limitations and potential local tissue irritation with topical use.

NAC has high aqueous solubility, which facilitates the development of concentrated oral, inhaled, and parenteral dosage forms; depending on the experimental conditions, solubility values ranging from approximately 100 to 163.9 mg/mL have been reported [55,56]. From a biopharmaceutical perspective, NAC has traditionally been classified as a BCS Class I drug based on its high solubility and presumed high intestinal permeability [57]; however, some data indicate that its classification between BCS Classes I and III remains inconclusive, because intestinal permeability may vary and may depend on biological factors, including the gut microbiota [56]. This uncertainty is directly relevant to formulation development: if systemic exposure is limited primarily by permeability rather than dissolution, further enhancement of solubility alone is unlikely to substantially improve oral bioavailability.

Chemical instability is also a serious problem: NAC is prone to oxidation, resulting in the formation of the disulfide N,N′-diacetyl-L-cystine (Di-NAC) and other degradation products, which may reduce the amount of chemically active reduced NAC and shorten the shelf life of formulations [58]. Therefore, exposure to oxygen, temperature, storage duration, and formulation composition are important determinants of NAC stability. At room temperature, 10.3–14.9% degradation was observed after 72 h, although the solutions remained within the predefined stability criterion for at least 60 h [59]. Liposomal formulations of NAC and azithromycin showed high instability in plasma and sputum (loss of >90% of the drug within 48 h) [42]. These findings emphasize the need to optimize carrier composition, protect NAC from oxidation, and control storage conditions.

Despite its high solubility, systemic exposure to unchanged NAC after oral administration remains low because of extensive presystemic metabolism; its absolute oral bioavailability is approximately 4–11% [60,61]. Low oral bioavailability is associated with extensive presystemic metabolism, including transformations in the intestine and liver, while ionization behavior and limitations in intestinal permeability may further affect absorption. Therefore, the route of administration and delivery system are important determinants of therapeutic exposure, and strategies aimed at increasing epithelial permeability, protecting NAC from presystemic metabolism, prolonging local retention, and improving tissue penetration may be more important than further increasing its solubility.

Additional factors limiting use include unpleasant organoleptic properties (bitter taste, astringent effect, and characteristic sulfurous odor), which complicate oral administration and reduce patient compliance [62], as well as strain specificity and dependence on the particular antibiotic—the efficacy of NAC varies by bacterial species and even strain, and synergy is not observed with all antibiotics of a given class [39,40].

These physicochemical and pharmacokinetic properties directly influence formulation development and the therapeutic efficacy of NAC. High aqueous solubility facilitates the preparation of concentrated formulations but does not itself overcome the problem of low systemic exposure; ionization and potential limitations in intestinal permeability may hinder absorption, while extensive presystemic metabolism further reduces oral bioavailability. At the same time, oxidation decreases the amount of chemically active reduced NAC, whereas the need to achieve high antibiofilm concentrations imposes additional requirements on the delivery system. These limitations are driving active development of new delivery systems and dosage forms capable of protecting NAC from oxidation, controlling local pH and drug release, increasing local retention and tissue penetration, and achieving high concentrations directly at the biofilm site while minimizing systemic exposure and local irritation.

### 8.1. Respiratory Infections and Cystic Fibrosis

Chronic respiratory infections, particularly in cystic fibrosis and chronic obstructive pulmonary disease, are characterized by viscous sputum with a high mucin content, which creates a physical barrier to antibiotic penetration. Biofilms of *P. aeruginosa* and other pathogens form in the airways, further limiting the effectiveness of antimicrobial therapy. Sputum viscosity is due mainly to disulfide bonds in mucin polymers—precisely the target effectively attacked by NAC, creating a rationale for its synergistic action with antibiotics, provided delivery is adequate. For respiratory application, the NAC delivery system must provide mucus penetration, protection of the thiol group from oxidation, prolonged action, and suitability for inhalation (aerodynamic particle size of 1–5 µm). Guerini et al. developed chitosan-based microstructured lipid carriers (MLC) containing NAC; the use of chitosan in the internal aqueous phase yielded particles with a narrow size distribution and high encapsulation efficiency [63]. NAC-loaded MLC showed higher antioxidant activity than the free molecule. Against *P. aeruginosa* biofilm growth, reduction was 64.74% ± 6.2% at 0.5 mg/mL and 83.74% ± 9.95% at 2 mg/mL, considerably lower than the effective concentrations of free NAC. Pinto et al. proposed an alternative approach based on encapsulating NAC in lipid nanoparticles (LNPs) functionalized with D-amino acids to target bacterial biofilms [64]. The optimized formulations had a mean hydrodynamic diameter of about 200 nm, a low polydispersity index, and high loading capacity; they were stable under storage for up to 6 months. Biocompatibility studies showed low cytotoxicity toward fibroblasts and low hemolytic activity, with NAC-loaded LNPs having a safer profile (less hemolysis) than empty LNPs. In combination with moxifloxacin, LNPs showed significant potential in reducing bacterial viability in *P. aeruginosa* biofilms. Aljihani et al. investigated the synergistic effect of liposomal formulations co-encapsulating azithromycin and NAC against resistant clinical strains of *E. coli*: liposomal azithromycin with NAC (LAN) reduced the MIC for *E. coli* strain SA10 to 2.5 µg/mL (compared with 16 µg/mL for free azithromycin) and provided a 93.2% reduction in biofilm at a concentration equal to the MIC [42]. However, the authors noted limited stability in sputum and plasma, indicating the need for further optimization. MLCs with chitosan and LNPs with D-amino acids appear most promising for respiratory infections, showing good efficacy in vitro and an acceptable safety profile, whereas liposomal formulations with azithromycin require further investigation.

### 8.2. ENT Practice

Chronic rhinosinusitis is characterized by persistent inflammation of the nasal and paranasal sinus mucosa, in which staphylococcal biofilms play a key pathogenic role. The accessibility of this region for local irrigation creates favorable conditions for topical NAC application. The pH of the nasal mucosa is typically 5.5–6.5, which may modulate NAC activity. Otitis, especially chronic otitis with tympanostomy tubes, creates an anatomical niche for biofilm formation on the surface of implanted devices. ENT applications require a high local NAC concentration, good mucosal adhesion, ease of use (sprays, drops, irrigation solutions), and minimal systemic absorption. In a prospective study involving 75 patients with chronic rhinosinusitis, Jotic et al. showed that a concentration of 3.1 mg/mL was sufficient to completely prevent biofilm formation in 77.8% of *S. aureus* isolates, while 49.6 mg/mL led to complete eradication of established biofilm in 81.5% of isolates [44]. Jun et al. investigated the inhibitory activity of NAC against biofilms on tympanostomy tubes, confirming that NAC can prevent colonization of medical devices in ENT practice [65]. Božić et al. investigated the effect of a fixed combination of NAC and dry propolis extract on bacterial pathogens isolated from upper respiratory tract infections; the combination showed a synergistic effect at all stages of biofilm formation, with a mean MIC of 1.87/0.187 mg NAC/mg propolis against *S. aureus* [66]. The data are most compelling for ENT practice: NAC irrigation solutions already demonstrate clinical efficacy, and combination with propolis represents a promising strategy for enhancing antibiofilm action.

### 8.3. Dentistry

Periodontal and endodontic infections are associated with the formation of multispecies biofilms in hard-to-reach periodontal pockets and root canals. The anaerobic environment, complex biofilm architecture, and limited drug access substantially reduce the effectiveness of standard antimicrobial therapy. Dental application requires deep penetration, antianaerobic activity, low toxicity to periodontal tissues, and compatibility with endodontic irrigation protocols. Moon et al. showed that NAC at a concentration of 0.78–1.56 mg/mL is effective against endodontic pathogens, including *Actinomyces naeslundii*, *Lactobacillus salivarius*, *Streptococcus mutans*, and *Enterococcus faecalis* [51]. Abu Hasna et al. demonstrated that NAC in combination with photodynamic therapy exhibits a synergistic effect against *E. faecalis* in root canals. NAC was more effective in eliminating multispecies endodontic biofilms than traditional medicaments, and 100 mg/mL NAC completely disrupted mature multispecies biofilms [52]. NAC shows promising results in dentistry, especially in endodontics, where its ability to disrupt multispecies biofilms may offer an alternative to traditional irrigants; combined approaches with photodynamic therapy are promising.

NAC may also be considered as an orthodontic material, as investigated in [67]. The combination of NAg and NAC exhibited marked antibacterial activity: the cement suppressed metabolic activity and lactic acid production in *Streptococcus mutans* biofilms, reduced the number of viable bacteria in the biofilm by 2 logarithmic orders (*p* < 0.05), and reduced protein adsorption by 76.87% (*p* < 0.05). At the same time, the cement retained acceptable biocompatibility and bond strength to enamel. This approach demonstrates the potential of NAC not only as a therapeutic agent but also as a component of preventive dental materials that can inhibit biofilm formation on the surfaces of orthodontic appliances and reduce the risk of enamel demineralization.

### 8.4. Urology

Catheter-associated urinary tract infections are among the most common nosocomial infections. *P. aeruginosa* is the third most common causative agent, responsible for 12% of all nosocomial urinary tract infections. Biofilms on catheter surfaces can reach a thickness of 500 µm and cause catheter occlusion through salt crystallization. Key requirements for urological application include prevention of bacterial adhesion, suppression of urease activity, and prolonged action under conditions of continuous urine flow. Manoharan et al. demonstrated that NAC significantly reduces the number of viable bacteria (>4 log10, *p* < 0.01) and inhibits biofilm formation and the associated catheter occlusion for up to 96 h. NAC also suppressed bacterial adhesion by modulating colloidal forces [19]. El-Feky et al. showed that catheters impregnated with a combination of ciprofloxacin and NAC had the greatest inhibitory effect on microbial adhesion from 85.5% to 100% against a wide range of pathogens [50]. Manoharan et al. also showed that NAC is a potent urease inhibitor that prevents the formation of crystalline biofilm [47]. In a clinical study by Singh et al., oral NAC (600 mg/day) completely prevented biofilm formation on urethral stents [48]. Cai et al. showed that adding NAC to fosfomycin in patients with chronic bacterial prostatitis achieved complete pathogen eradication in 75% of patients [43]. NAC catheter coatings represent the most clinically substantiated strategy in urology.

### 8.5. Gynecology

Bacterial vaginosis is characterized by disruption of the vaginal microbiota with predominance of *Gardnerella vaginalis*. Biofilms are observed in 90% of patients. The vaginal environment has an acidic pH (3.5–4.5), which is ideal for NAC activity. This physiological advantage could be exploited to develop preparations active specifically in an acidic environment. Despite the biological rationale, specific studies of NAC delivery systems for gynecological applications are extremely scarce in the published literature. In a systematic review, Dinicola et al. noted that study findings support the potential role of NAC as an adjuvant molecule in the treatment of bacterial biofilms, with an excellent safety profile across various body sites, including the vaginal cavity [68]. The gynecological niche represents an understudied but highly promising direction for the development of innovative dosage forms.

### 8.6. Wound Infections and Diabetic Ulcers

Chronic wounds, particularly diabetic foot ulcers, are characterized by prolonged inflammation, oxidative stress, and the presence of biofilms. The economic burden of all chronic non-healing wounds in the United States is estimated at more than $50 billion per year, and the 5-year mortality rate for diabetic foot ulcers (30.5%) is comparable to that of some malignant neoplasms. Pranantyo et al. developed a hydrogel dressing with dual antibiofilm and antioxidant functionality, based on a cross-linked polyethylene glycol network with a covalently attached antibacterial cationic polyimidazolium and antioxidant NAC [54]. A key advantage is that NAC is chemically bound to the hydrogel matrix (low-leaching), providing prolonged action without wound contamination. In a mouse model of diabetic wounds, the hydrogel accelerated wound closure, and in a three-dimensional ex vivo human skin equivalent model, NAC stimulated keratinocyte differentiation and re-epithelialization [54]. Vishnevetskii et al. developed a one-pot synthesis of sols and hydrogels containing silver nanoparticles and nanoclusters, using only NAC as the reducing, stabilizing, and gelling agent [69]. The resulting NAC-AgNC systems, at concentrations of 20–40 µM, completely inhibited biofilm formation by *A. baumannii* and *P. aeruginosa*, showing twice the activity of L-cysteine-based systems, while being non-toxic to normal human cells. Hydrogels with immobilized NAC and silver-based nanosystems represent the most mature and clinically relevant strategies for wound treatment.

### 8.7. Bone Infections

Osteomyelitis is one of the most common complications in orthopedic surgery, caused mainly by *S. aureus.* Bacterial biofilm formation renders systemic antibiotic therapy ineffective. Polo-Montalvo et al. proposed a nanosystem based on mesoporous bioactive glass nanoparticles (MBGN) with a composition of 82.5 SiO_2_–17.5 CaO, loaded with levofloxacin and functionalized with NAC [70]. The MBGN provided levofloxacin loading of up to 23.1%; NAC was covalently bound while preserving an accessible thiol group. The combination of NAC and levofloxacin enhances activity against *S. aureus* by disrupting the integrity of the mature biofilm. The nanosystem was biocompatible with pre-osteoblasts, enhanced their differentiation, and promoted biomimetic mineralization in vitro [70]. The MBGN-L-NAC nanosystem represents an innovative approach combining the antibiofilm action of NAC with the osteoinductive properties of bioactive glass.

Table 6 summarizes the key strategies for each clinical area.

### 8.8. Challenges of Clinical Translation and Future Directions

Despite promising results, significant barriers remain to the clinical implementation of NAC delivery systems, which can be systematized into three categories: technological, regulatory, and biological. Scalability and compliance with Good Manufacturing Practice (GMP) standards for many nanoscale systems remain unresolved issues; the production of LNPs, MLCs, and MBGNs requires sophisticated equipment, strict quality control, and process reproducibility, which increases the cost of the final product and limits its accessibility. The development of simple, scalable synthesis methods, such as the one-pot synthesis of Vishnevetskii et al. [69], may help overcome this barrier. The absence of standardized protocols for evaluating combined nanopreparations creates obstacles to their registration; NAC-containing nanosystems require the development of specific pharmacopeial quality control methods, including assessment of loading, stability, release profile, and biocompatibility. The European Medicines Agency (EMA) and the Food and Drug Administration (FDA) currently have no specific guidelines for combined “antibiotic + antibiofilm agent” preparations, which slows their market entry. The long-term toxicity of nanocarriers and their in vivo metabolism remain insufficiently studied; although NAC-loaded LNPs showed a safer profile than empty nanoparticles [64], and NAC-AgNC systems were non-toxic to human cells [69], systematic studies of chronic toxicity and tissue bioaccumulation are lacking. Biofilm heterogeneity is another concern: efficacy demonstrated in laboratory strains may not be reproduced in clinical isolates, underscoring the need for testing on clinical material and a personalized approach. Successful clinical translation over the next 5–10 years will require randomized controlled trials with large sample sizes to confirm efficacy and safety, the development of standardized protocols for evaluating the antibiofilm activity of nanosystems, studies of stability and shelf life under various storage conditions, toxicological studies in animals with long-term follow-up, the development of commercially viable and scalable manufacturing processes, and the creation of regulatory guidelines for combined antibiofilm preparations.

## 9. Future Directions for NAC Research

Further investigation of NAC as an antibiofilm agent should be oriented towards the integration of computational methods, advanced drug delivery systems, multi-omics analysis, and rapid diagnostics. Such an approach is particularly important given the instability of NAC, its limited bioavailability, and the pH dependence of its activity, all of which may reduce the reproducibility of its effects in vivo.

### 9.1. Artificial Intelligence for Screening Combinations

Machine learning methods can accelerate the search for synergistic combinations of NAC with antibiotics by simultaneously analyzing a large number of parameters, including the mechanism of action of the antibiotic, the resistance profile of the pathogen, minimum inhibitory concentrations, biofilm age, extracellular matrix composition, pH, and culture conditions. Classification models can predict the likelihood of synergy, while regression algorithms can identify optimal component ratios and concentration ranges. Active learning, in which the algorithm selects the most informative combinations for experimental validation, is a particularly promising direction. This approach can reduce the number of empirical experiments required and uncover combinations whose effectiveness depends on the specific microbial species or biofilm structure. However, computational predictions should not be viewed as a substitute for experimental validation. Standardized methods for biofilm assessment, independent confirmation of results, and consideration of differences between laboratory models and clinical isolates remain essential.

### 9.2. Optimization of Dosage Forms

Nanoparticles, liposomes, and hydrogels can be used to protect NAC from degradation, increase its local concentration, and enable controlled release at the site of infection. Machine learning can link formulation composition and manufacturing parameters to particle size, polydispersity, surface charge, encapsulation efficiency, stability, and release kinetics. For NAC, the development of systems that preserve the drug during storage and release it under conditions characteristic of the biofilm microenvironment is of particular importance. Models must account for pH-dependent behavior, possible oxidation, interactions with matrix components, and compatibility with antibiotics. Multi-objective algorithms that simultaneously optimize loading capacity, stability, biocompatibility, and antibiofilm activity are especially promising. Stimulus-responsive carriers that release NAC in response to changes in pH, the presence of enzymes, or reducing conditions represent a rational direction for development. Such systems could enhance the selectivity of delivery and minimize drug loss prior to contact with the biofilm.

### 9.3. Multi-Omics Analysis

The combined application of metagenomics, transcriptomics, proteomics, and metabolomics can provide a detailed understanding of NAC’s action at multiple levels of biofilm organization. Metagenomic analysis can characterize the composition of the microbial community and resistance genes. Transcriptomics can assess changes in the expression of genes involved in adhesion, matrix synthesis, quorum sensing, and stress responses. Proteomics can reveal rearrangements in surface proteins and enzyme systems, while metabolomics can track alterations in energy metabolism, redox status, and the production of signaling molecules. Time-series analysis is of particular value, as it allows the differentiation between primary matrix disruption, subsequent bacterial cell damage, and adaptive responses of surviving microorganisms. Such data could help determine whether NAC should be administered before, simultaneously with, or after antibiotic treatment.

### 9.4. Precision and Phage Therapy

Precision antimicrobial therapy should take into account the genetic profile of the pathogen, phenotypic susceptibility, microbial community composition, and the physical characteristics of the biofilm. On this basis, it would be possible to select the appropriate antibiotic, NAC dose, dosage form, route of administration, and treatment duration. The combination of bacteriophages with NAC-containing nanoparticles represents a particularly intriguing avenue. NAC can facilitate phage penetration through the biofilm matrix, while phages provide highly specific targeting of bacterial cells. Nanoparticles have the potential to simultaneously protect both NAC and phage particles, ensuring their co-delivery. Key unresolved questions include the preservation of phage infectivity, formulation stability, immunological responses, and the potential for bacterial adaptation to phage exposure.

### 9.5. Personalized Management

A forward-looking treatment model would combine rapid pathogen identification, resistance gene detection, biofilm assessment, and regular monitoring of treatment response. An initial regimen could be prescribed based on rapid diagnostic results and subsequently adjusted according to microbial burden, clinical dynamics, and the emergence of resistant variants. The future of NAC as an antibiofilm agent lies in the transition from non-specific application to targeted, context-dependent therapy. The integration of artificial intelligence, optimized delivery systems, multi-omics analysis, phage technologies, and rapid diagnostics has the potential to compensate for the instability, limited bioavailability, and pH dependence of NAC, thereby improving the predictability of its effects and expanding its clinical utility.

## 10. Conclusions

*N*-acetylcysteine, initially known as a mucolytic agent, is now regarded as a multifunctional antibiofilm agent with proven potential in the adjuvant therapy of biofilm-associated infections. This review systematizes the data accumulated by 2026 on its mechanisms of action, synergistic combinations with antibiotics, clinical studies, and promising delivery strategies.

NAC has a multitarget action that includes disruption of matrix proteins and polysaccharides, degradation of extracellular DNA, suppression of quorum sensing, and disturbance of intracellular redox balance. This favorably distinguishes NAC from many other antibiofilm agents that act on only a single matrix component. The ability of NAC to enhance the activity of antibiotics (particularly β-lactams, polymyxins, tetracyclines, and macrolides) opens up possibilities for treating infections caused by multidrug-resistant strains.

Clinical studies confirm the efficacy of NAC as an adjuvant in urinary tract infections, chronic rhinosinusitis, diabetic osteomyelitis, and cystic fibrosis. However, widespread implementation is constrained by pH dependence, instability, low bioavailability, and the need for high concentrations. Innovative delivery systems: nanoformulations (LNPs, MLCs, MBGNs), hydrogel systems, and combinations with propolis or chitosan demonstrate the ability to overcome these limitations, reducing effective concentrations and increasing clinical efficacy. Systems in which NAC serves not only as an active agent but also as a structural component (NAC-AgNC systems, hydrogels with covalently attached NAC) deserve particular attention, as they expand the functional capabilities of materials.

Further research should focus on conducting randomized controlled trials with large sample sizes, developing standardized protocols for topical application, creating stable combined preparations for systemic and local therapy, and overcoming the technological and regulatory barriers to clinical translation. Hydrogels for wound treatment and catheter coatings appear the most substantiated for near-term implementation, whereas nanoformulations for respiratory and bone infections require further preclinical and clinical study.

## Figures and Tables

**Table 1:** Main biofilm-forming pathogens and typical sites of infection.

Pathogen	Infection/Site	Source
*Mycobacterium abscessus*, *Haemophilus influenzae*	Cystic fibrosis	[9]
*Borrelia burgdorferi*	Infected skin lesions	[9]
*P. aeruginosa*	Bronchiectasis	[9]
*S. aureus*, *P. aeruginosa*	Chronic wound infections	[9]
*E. coli*	Urinary tract infections	[9]
*S. aureus*, *S. epidermidis*	Infective endocarditis	[9]
*Gardnerella vaginalis*	Bacterial vaginosis	[11]
*H. influenzae*, *Moraxella catarrhalis*, *Streptococcus pneumoniae*	Otitis media	[11]
*P. aerobicus*, *Fusobacterium nucleatum*	Periodontitis	[10]
*P. aeruginosa*	Osteomyelitis	[10]
*Vibrio cholerae*	Cholera	[3]
*S. aureus*, *S. pneumoniae*, *H. influenzae*, *M. catarrhalis*	Chronic rhinosinusitis	[3]
*S. aureus*, *H. influenzae*, *C. albicans*, *M. nonliquefaciens*, *Propionibacterium acnes*	Pharyngitis, laryngitis	[3]
*E. faecalis*, *Staphylococcus* spp., *E. coli*	Chronic bacterial prostatitis	[3]
*A. baumannii*, *H. influenzae*	Meningitis	[3]
*Burkholderia pseudomallei*	Melioidosis	[12]

**Table 2:** Comparison of NAC with other chemical antibiofilm agents.

Agent	Strengths	Weaknesses
*N*-acetylcysteine	Multiple mechanisms; synergy with antibiotics; no direct resistance at low doses; activity against mature biofilms	pH dependence (effective at pH < pKa); high concentrations required for full effect; strain specificity; weak direct anti-QS activity
EDTA	Effective against Gram-negative bacteria; enhances antibiotic penetration; fast action	Weak activity against Gram-positive bacteria; tissue demineralization; high concentrations required; systemic toxicity
Silver (nanoparticles)	Broad spectrum; synergy with EDTA; activity against Gram-positive and Gram-negative bacteria; low resistance rate; prolonged activity	Cytotoxicity at high concentrations; allergy risk; interaction with chlorides; high cost
Chlorhexidine	Long-lasting tissue binding; broad spectrum, including fungi	Ineffective against mature biofilms; mucosal irritation; bacterial enzymes reduce activity
Hydrogen peroxide	Fast action; low cost	Ineffective against mature biofilms; instability; tissue irritation; only a statistical reduction in biofilms
DNase	Prolonged inhibition of formation; enhances antibiotic effect; synergy with chitosan	Limited efficacy as monotherapy; high cost; sensitivity to proteases
Chitosan	Synergy with antibiotics; fast action; up to 95% efficacy against mature biofilms; biocompatibility; biodegradation	Dependence on degree of polymerization; solubility only under acidic conditions; limited penetrating ability as monotherapy
Lactoferrin	Disrupts existing biofilms; biocompatibility; immunomodulation; broad spectrum	High cost; mechanism not fully understood; sensitivity to proteolysis; variable efficacy
Povidone-iodine	Effective in combination with H_2_O_2_; broad spectrum; fast action; low toxicity	Tissue staining; irritation; contraindicated in iodine allergy; effect on the thyroid gland; ineffective as monotherapy against mature biofilms

**Table 3:** Synergy of NAC with antibiotics of various classes.

Antibiotic Class	Antibiotic	Interaction	Bacterial Strain	Ref.
Carbapenems	Meropenem	Synergy	*K. pneumoniae*, *A. baumannii*	[39]
	Imipenem	Antagonism	*P. aeruginosa*	[39]
Penicillins	Ampicillin/sulbactam	Synergy	*K. pneumoniae*, *A. baumannii*	[39]
	Penicillin	Partial synergy	*Corynebacterium striatum*	[39]
	Amoxicillin/clavulanate	Partial synergy	*C. striatum*	[39]
	Piperacillin/tazobactam	Partial synergy	*K. pneumoniae*	[39]
Cephalosporins	Cefditoren	Synergy	*S. pneumoniae*	[40]
Aminoglycosides	Tobramycin	Synergy	*P. aeruginosa*	[21]
	Amikacin	Moderate synergy	Not specified	[21]
	Gentamicin	Variable synergy	Gram-negative	[21]
Fluoroquinolones	Ciprofloxacin	Weak synergy	*P. aeruginosa*	[13]
Polymyxins	Colistin	Synergy	*P. aeruginosa*	[30]
Glycylcyclines	Tigecycline	Synergy	*A. baumannii*, MRSA, *S. epidermidis*	[41]
	Azithromycin (free)	Synergy	*E. coli*	[42]
Macrolides	Azithromycin (liposomal)	Synergy	*E. coli*, *P. mirabilis*, *S. aureus*, *E. faecalis*, *P. aeruginosa*	[42]
Phosphonic acids	Fosfomycin	Synergy	*E. coli*, *P. mirabilis*, *S. aureus*, *E. faecalis*, *P. aeruginosa*	[43]
Oxazolidinones	Linezolid	Variable	*C. striatum*, *S. aureus*	[39]
Lincosamides	Clindamycin	Variable	*C. striatum*	[39]
Sulfonamides	Trimethoprim/sulfamethoxazole	Variable	*C. striatum*	[39]

**Table 4:** Key clinical studies of the antibiofilm efficacy of NAC (2016–2026).

Pathogen	Infection/Site	NAC Concentration	Effect/Outcome	Study Design, Duration, Cohort	Ref.
*Streptococcus faecalis*, *Clostridium butyricum*, *Bacillus mesentericus*	Urinary tract infections	600 mg/day (oral)	Reduction in recurrences	Open-label pilot; 16-day regimen + 6-month follow-up; *n* = 28 men with difficult-to-treat chronic bacterial prostatitis (67.8% ESBL); pathogens included typical uropathogens	[43]
*E. coli*, *Klebsiella oxytoca*	Chronic prostatitis	600 mg/day (oral) + fosfomycin	Complete eradication in 75%	Open-label, single-arm pilot study; 16-day treatment (FT 3 g q48h after 2-day loading + NAC 600 mg daily for 2 weeks) with 6-month follow-up; 28 men with difficult-to-treat chronic bacterial prostatitis (67.8% ESBL)	[43]
*S. aureus* (incl. MRSA)	Chronic rhinosinusitis	3.1–49.6 mg/mL (irrigation)	Inhibition of formation in 77.8%, eradication of mature biofilms in 81.5%	Prospective in vitro study; single time-point testing of NAC concentrations; 75 patients with chronic rhinosinusitis (CRSwNP/CRSsNP), 54 Staphylococcal isolates from sinonasal tissue.	[44]
*S. aureus*, *P. aeruginosa*	Diabetic foot osteomyelitis	600 mg twice daily (oral) + antibiotics	Accelerated clinical response, decreased CRP	Open-label RCT; 2-week NAC regimen (600 mg BID) with 3-week follow-up; 53 adults with Wagner grade III–IV diabetic foot osteomyelitis	[45]
*H. pylori*	Gastric mucosa	600 mg/day	600 mg/day	Randomized, double-blind, placebo-controlled trial; 2-week treatment (NAC 600 mg BID + triple therapy) with 2-month follow-up; *60* adults with dyspepsia and *H. pylori* infection	[46]
*S. epidermidis*	Chronic rhinosinusitis	2.5–10 mg/mL	Biofilm disruption	In vitro experimental study; single time-point testing of *NAC* concentrations; clinical *P. mirabilis* isolates and bladder epithelial cell lines (no patient cohort)	[47]
*S. aureus*, *S. epidermidis*	Hemodialysis catheter	600 mg twice daily	Reduced colonization	Prospective, randomized, controlled pilot study; 5-week treatment (NAC 600 mg BID vs. probiotics) with assessments at stent removal; 60 adults undergoing ureteral stent placement	[48]
*S. aureus*, *S. pyogenes*, *S. pneumoniae*, *H. influenzae*	Upper respiratory tract infections	As part of combination therapy	Clinical improvement	Open-label, prospective, sequential-therapy study; 10-day regimen (day 1 IM thiamphenicol–NAC, days 2–10 aerosol) with 15-day follow-up; 102 adults with recurrent URTIs (24 with in vivo biofilm-positive infections)	[49]

**Table 5:** Key preclinical studies.

Pathogen	Model	NAC Concentration	Effect/Outcome	Ref.
*P. aeruginosa*	Cystic fibrosis (in vitro)	8 mg/mL (inhalation) + colistin	Complete eradication of biofilms	[39]
*P. mirabilis*	Bladder, catheter (in vitro)	1 mM (0.16 mg/mL)	Prevention of catheter occlusion	[50]
*S. aureus*, *S. epidermidis*, *E. coli*, *K. pneumoniae*, *P. aeruginosa*, *P. vulgaris*	Ureteral stent (irrigation, in vitro)	2–4 mg/mL	Prevention of biofilm formation	[50]
*Actinomyces naeslundii*, *Lactobacillus salivarius*, *S. mutans*, *E. faecalis*	Endodontic infections (in vitro)	0.78–1.56 mg/mL	Antibacterial effect	[51,52]
*P. acnes*, *E. faecalis*, *S. pyogenes*, *C. ammoniagenes*	Skin infections (in vitro)	12.5 mg/mL	Inhibition of growth and adhesion	[53]
MRSA, *P. aeruginosa*	Infected diabetic wounds (mice)	0.2 mg (topical, in hydrogel)	Accelerated healing	[54]

**Table 6:** Optimal NAC delivery systems for various clinical niches.

Clinical Area	Main Challenge	Optimal Delivery System	Key Advantage	Refs.
Respiratory (CF, COPD)	Mucus penetration, instability	Chitosan-based MLC; LNPs with D-amino acids	Biofilm reduction of 83.7% at 2 mg/mL; safety	[63,64]
ENT (rhinosinusitis, otitis)	Local action, compliance	Irrigation solutions; combination with propolis; tube coatings	Biofilm eradication in 81.5% of isolates; prevention of tube colonization	[44,65,66]
Dentistry	Deep penetration, anaerobes	Irrigation; combination with photodynamic therapy	Complete disruption of multispecies biofilms at 100 mg/mL	[51,52]
Urology (CA-UTI)	Prevention of biofilms on devices	Catheter coatings (NAC + ciprofloxacin)	Reduced adhesion by 85.5–100%; urease inhibition	[19,50]
Gynecology (BV)	Acidic environment, preservation of microflora	Vaginal gels, suppositories (promising niche)	Utilization of physiological pH; preservation of lactoflora	[68]
Wounds (diabetic ulcers)	Oxidative stress, regeneration	Hydrogels with immobilized NAC; AgNC-NAC systems	Accelerated healing; complete inhibition at 20–40 µM; non-toxicity	[54]
Bone infections	Osseointegration, local delivery	MBGN with NAC and levofloxacin	Enhanced antibiofilm effect, osteoinduction	[70]

## Data Availability

No new data were created or analyzed in this study. Data sharing is not applicable to this article.

## Abbreviations

The following abbreviations are used in this manuscript:

NAC	*N*-acetylcysteine
DNAC	Dendrimer *N*-acetylcysteine
Di-NAC	N,N′-diacetyl-L-cystine
LAN	Azithromycin with NAC
PRISMA	Preferred Reporting Items for Systematic reviews and Meta-Analyses
GRADE	Grading of Recommendations, Assessment, Development and Evaluation
MIC	Minimum Inhibitory Concentration
QS	Quorum Sensing
RCT	Randomized Controlled Trial
LAN	Liposomal Azithromycin with NAC
COPD	Chronic Obstructive Pulmonary Disease
CF	Cystic Fibrosis
CA-UTI	Catheter-Associated Urinary Tract Infection
BV	Bacterial Vaginosis
MRSA	Methicillin-Resistant *Staphylococcus aureus*
GMP	Good Manufacturing Practice
FDA	Food and Drug Administration
EMA	European Medicines Agency
MLC	Microstructured Lipid Carriers
LNPs	Lipid Nanoparticles
MBGN	Mesoporous Bioactive Glass Nanoparticles
CRP	C-reactive protein
NAg	Silver Nanoparticles
AHL	Acyl-Homoserine Lactone
DNA	Deoxyribonucleic Acid
ROS	Reactive Oxygen Species
EPS	Extracellular Polymeric Substances
LPS	Lipopolysaccharide
NET	Neutrophil Extracellular Trap
BCS	Biopharmaceutics Classification System
